# Predominant Role of Plasmacytoid Dendritic Cells in Stimulating Systemic Autoimmunity

**DOI:** 10.3389/fimmu.2015.00526

**Published:** 2015-10-12

**Authors:** Xinfang Huang, Stephanie Dorta-Estremera, Yihong Yao, Nan Shen, Wei Cao

**Affiliations:** ^1^Shanghai Institute of Rheumatology, Shanghai Renji Hospital, Shanghai Jiaotong University School of Medicine, Shanghai, China; ^2^Department of Immunology, The University of Texas MD Anderson Cancer Center, Houston, TX, USA; ^3^The University of Texas Graduate School of Biomedical Sciences, Houston, TX, USA; ^4^Cellular Biomedicine Group Inc., Palo Alto, CA, USA

**Keywords:** plasmacytoid dendritic cells, systemic lupus erythematosus, type I interferon, amyloid, autoantibody, autoimmunity, animal models, B cells

## Abstract

Plasmacytoid dendritic cells (pDCs), which are prominent type I interferon (IFN-I)-producing immune cells, have been extensively implicated in systemic lupus erythematosus (SLE). However, whether they participate critically in lupus pathogenesis remains unknown. Recent studies using various genetic and cell type-specific ablation strategies have demonstrated that pDCs play a pivotal role in the development of autoantibodies and the progression of lupus under diverse experimental conditions. The findings of several investigations highlight a notion that pDCs operate critically at the early stage of lupus development. In particular, pDCs have a profound effect on B-cell activation and humoral autoimmunity *in vivo*. This deeper understanding of the vital role of pDCs in lupus pathogenesis supports the therapeutic targeting of the pDC-IFN-I pathway in SLE.

## Introduction

Patients with systemic lupus erythematosus (SLE) frequently have aberrant expression of genes that are stimulated by type 1 interferons (IFN-α, IFN-β, IFN-ω, IFN-τ; IFN-I), a family of pluripotent cytokines that are important for antiviral immune response, and this expression profile is correlated with anti-dsDNA antibody levels and disease severity (1, 2). Plasmacytoid dendritic cells (pDCs), a distinct subset of DCs, are capable of rapidly secreting large amounts of IFN-I in response to viral infection through endosomal Toll-like receptor (TLR) activation. In SLE patients, pDCs are believed to be a major cellular source of IFN-I, primarily because they readily produce IFN-I when exposed to SLE immune complexes or other lupus-related, nucleic acid-containing compounds (3–5). The implications IFN-I and pDCs have in clinical SLE have been extensively investigated and reviewed previously (3, 6–8).

In this perspective, we focus on the findings of recent studies that collectively illuminate the involvement of pDCs in systemic autoimmunity *in vivo* and their role in promoting SLE through IFN-I production in particular. Studies using various experimental lupus models have revealed that pDCs play an indispensable role in stimulating autoantibody response and facilitating lupus progression, which bolsters the rationale of targeting pDCs to alleviating SLE.

## pDCs are Instrumental to Spontaneous Lupus Development

The development of pDCs from bone marrow progenitors is critically controlled by the transcription factors E2-2 (encoded by *Tcf4*) (9) and IRF8 (10, 11). In humans, naive pDCs, a distinct type of immune cells, abundantly express the cell type-specific receptor BDCA2, and in mice, they express PDCA1 and Siglec-H (3). Slc15a4, a peptide/histidine transporter, specifically facilitates endosomal TLR signaling and the production of IFN and other cytokines in pDCs (12). On the basis of these insights, researchers have developed several genetic systems for selectively deleting or disabling pDCs *in vivo* (Table 1). Consequently, several groups have investigated the contribution of pDCs to SLE in experimental lupus models.

**Table 1:** **Effects of pDC depletion in different spontaneous lupus models**.

Lupus model	Genetic alteration	Phenotypes elicited	Age of effect	ISG affected	B cells affected	Reference
NZB	*Irf8^−/−^*	Reduced anti-chromatin, anti-red blood cells, and anti-nuclear antibodies; decreased kidney disease; diminished splenomegaly	Constitutive	Unknown	Reduced CD21*^−^*CD23*^−^* subset	(13)
B6-*Faspr^lpr^*	*Slc54a2eeble^feeble^*	Prolonged survival; reduced anti-chromatin and anti-nuclear antibodies; decreased hypergammaglobulinemia; diminished splenomegaly and lymphadenopathy	Constitutive	Unknown	Reduced CD21*^−^*CD23*^−^* subset	(13)
*Tlr7*-tg	*Tcf4*^+/^*^−^* (global and CD11c-specific)	Prolonged survival; decreased splenomegaly; abolished myeloid cell expansion; reduced anti-RNA antibody; decreased kidney inflammation and IgG deposition in glomeruli	Constitutive	Unknown	Unknown	(14)
B6.Sle1.Sle3	*Tcf4*^+/^*^−^*	Decreased splenomegaly; reduced anti-dsDNA, anti-RNA, and anti-nuclear antibodies; decreased kidney inflammation and IgG deposition in glomeruli	Constitutive	Sca-1	Reduced spontaneous GCs and GC-related gene expression	(14)
BXSB male	*BDCA2-DTR* + DT	Lessened splenomegaly; reduced kidney inflammation; decreased anti-histone and anti-nuclear antibodies; transient decline of hypergammaglobulinemia and anti-histone, anti-La, anti-dsRNA, and anti-RNP antibodies	8 weeks	Yes (blood and kidney)	Reduced ABC subset, increased MZ and T1 B cells	(15)
B6.Nba2	*BDCA2-DTR* + DT	Decreased IgG deposition in glomeruli; reduced anti-chromatin IgG; decreased hypergammaglobulinemia and splenomegaly in young mice	4 weeks; 12 weeks	Yes	Reduced spontaneous GCs, plasma cells, IL-21, and increased MZ B cells in young mice only	(16)

*ISG, interferon-stimulated genes; GC, germinal center; ABC, age-related B cell; MZ, marginal zone; T1, transitional 1*.

New Zealand Black (NZB) mice spontaneously develop elevated immunoglobulin levels, anti-DNA antibodies, hemolytic anemia, and circulating immune complexes that cause glomerulonephritis. Baccala et al. reported that *Irf8* deficiency effectively abolished all the key autoimmune phenotypes in NZB mice, indicating that the IRF8-instructed program is essential to lupus development (13).

Mice homozygous for the lymphoproliferation spontaneous mutation (Fas*^lpr^*) show systemic autoimmunity, which is associated with glomerulonephritis and lymphadenopathy. pDC-defective C57BL/6-*Fas^lpr^* mice, which were generated by introducing the *Slc15a4* mutation that selectively disrupts the pDC-mediated TLR response (13), have reduced autoantibodies and splenomegaly and prolonged survival compared with pDC-intact mice. Despite these intriguing observations, pDCs’ influence on lupus pathogenesis remains uncertain because both *Irf8* and *Slc15a4* affect the development or function of other immune cell types (13, 17–20).

Monoallelic loss of *Tcf4* is sufficient to impair the innate immune function of pDCs in mice and humans (9). Sisirak et al. (14) examined the effects of *Tcf4* haplodeficiency in autoimmune mice overexpressing the endosomal RNA sensor *Tlr7* (*Tlr7*-tg) (21–23). Intriguingly, they found that both global and DC-specific *Tcf4* haplodeficiency abolished splenomegaly and myeloid cell expansion and diminished anti-RNA autoantibody levels in the presence of *Tlr7* overexpression, which suggests a profound involvement of pDCs in this lupus model.

B6.*Sle1.Sle3* mice contain genomic intervals of two susceptibility loci from the lupus-prone NZM2410 strain on a C57BL/6 background (24). They spontaneously develop glomerulonephritis and high titers of autoantibodies against dsDNA and chromatin. Similar to *Tlr7*-tg, *Tcf4* haplodeficiency effectively blocked the development of lupus, as indicated by the significantly reduced anti-DNA antibody levels and glomerulonephritis (14). Although *Tcf4* also affects the development of B and T cells as well as a subset of conventional DCs (cDCs) (25–27), these data nevertheless suggest that pDCs are critically involved in lupus pathogenesis and autoantibody production.

In transgenic mice that express the diphtheria toxin (DT) receptor (DTR) under the control of the *BDCA2* promoter, the administration of DT results in the selective and transient ablation of pDCs (28). BXSB is a recombinant inbred lupus-prone strain, in which male mice harbor the Y-linked autoimmune accelerator locus, which has a duplicated chromosome segment containing *Tlr7* (21–23). After successfully backcrossing the BDCA2-DTR transgene into a BXSB background, Rowland et al. treated pre-autoimmune male BXSB.DTR mice with continuous DT injections for 3 weeks (15). Depletion of pDCs reduced the activation and expansion of immune cells, restricted autoantibody production, and minimized kidney inflammation. DT treatment also had a lasting regulatory effect on the serum levels of inflammatory factors, such as soluble VCAM-1, soluble CD40L, and chemokines (15). The finding that an early and transient depletion of pDCs was sufficient to ameliorate lupus further highlighted the predominant role pDCs have in lupus pathogenesis.

In another study, Davison and Jorgensen assessed the impact of transient pDC depletion in B6.Nba2 mice, which harbor IFN^+^ pDCs and develop spontaneous lupus-like disease (16). Nba2.BDCA2-DTR mice received continuous DT treatment, which significantly decreased antichromatin autoantibody levels and diminished the deposition of IgG immune complexes in kidney glomeruli.

Collectively, these studies demonstrate that pDCs play a pivotal role in promoting lupus-like autoimmunity in mice with diverse genetic backgrounds.

## Activation of pDCs and Induction of IFN-I Instigate Humoral Autoimmunity

The identical twin concordance rate for lupus is less than 50% (29, 30), which suggests that factors in addition to genetics also control SLE. Indeed, infections, drugs, and chemical compounds have been shown to facilitate the development of systemic autoimmunity (29, 31–33). However, much less is known about the molecular determinants and cellular pathways critical to this process.

One particular agent implicated in systemic autoimmunity recently is terminally misfolded amyloid proteins, which contain extensive β-sheet structures (34, 35). More than two dozen aberrant polypeptides that deposit amyloid have been implicated in human pathological conditions, including Alzheimer disease, Parkinson disease, and type 2 diabetes (35–38). In human tissues, amyloid often contains non-proteinaceous cofactors (34, 39) because amyloid precursor proteins have an affinity for nucleic acids and glycosaminoglycans, and interactions with these molecules expedite the formation of amyloid (40). Intriguingly, nucleic acid-containing amyloid fibrils activate endosomal TLRs to induce the release of IFN-I from pDCs (41). Our group showed that injections of DNA-containing amyloid induced pDC infiltration and IFN-I production in mice. We also found that the inoculation of non-autoimmune mice with DNA-containing amyloid stimulated the development of antinuclear autoantibodies and led to the deposition of immunoglobulin G in the kidney glomeruli (41). The findings of recent studies of amyloid-triggered autoimmunity in mice are summarized in Table 2. Importantly, the transient antibody-mediated depletion of pDCs prior to amyloid inoculation abolished IFN-I production and subsequent autoantibody generation. These findings illustrate that the innate immune activation of pDCs can break immune tolerance and initiate autoimmune responses. Given that pDCs actively secrete IFN-I during the early stages of many viral infections (3, 28), the pDC-IFN-I pathway may also contribute to the production of broadly self-reactive antibodies that are aggravated by viral infections.

**Table 2:** **Amyloid-triggered autoimmunity in mice**.

Mouse strain	Stimulating agent	Phenotype induced	IFN	pDC involvement	Reference
Balb/c	DNA-amyloid (mammalian)	Anti-histone, anti-ssDNA, anti-smRNP, and anti-nuclear antibodies; proteinuria, and IgG deposition in glomeruli	Yes	Yes	(41)
C57BL/6	DNA-amyloid (bacterial)	Anti-dsDNA and anti-chromatin antibodies	Yes	Unknown (cDCs produce IFN)	(42)
NZB/W F1	DNA-amyloid (bacterial), curli^+^ *E. coli*, curli^+^ *Salmonella*	Anti-dsDNA, anti-chromatin, and anti-nuclear antibodies	Yes	Unknown (cDCs produce IFN)	(42)
*Sle1,2,3*	DNA-amyloid (bacterial)	IgG2a and IgG2b subtypes of autoantibodies	Yes	Unknown (cDCs produce IFN)	(42)

Amyloid is not rare or intrinsically harmful. More prevalent than previously thought, functional amyloids participate in diverse cellular processes, including biofilm and spore assembly in bacteria, the storage of peptide hormones within mammalian secretory granules, and enhanced HIV infectivity during sexual transmission (43–48). Gallo et al. reported recently that curli, an amyloid expressed by *Enterobacteriaceae*, could irreversibly form fibers with bacterial DNA during biofilm formation. Curli-DNA composites stimulated high IFN-I and cytokine production in DCs *in vitro*. Interestingly, a bacterial amyloid–DNA complex and curli^+^ *Escherichia coli* and *Salmonella* triggered the production of autoantibodies in wild-type and lupus-prone mice (42). Although Gallo et al. did not analyze pDCs, DNA-containing bacterial amyloid shares key innate immune properties with mammalian amyloid. Therefore, the IFN-I induced by certain bacterial infections may also evoke humoral autoimmunity, an intriguing notion requiring further investigation.

## Mechanism of pDC-Mediated Autoimmune Responses

The functional contribution of pDCs to the pathogenesis of chronic diseases has been difficult to establish because (1) pDCs are rare immune cells; (2) IFN-I production by pDCs is rapid but transient; and (3) activated pDCs, by downregulating specific surface receptors, are almost indistinguishable from cDCs. Fortunately, new genetic tools are enabling researchers to elucidate the mechanism by which pDCs impact lupus development.

First, pDCs play a crucial role in the initiation of lupus. In pre-autoimmune BXSB mice, pDC depletion rapidly and effectively diminished hypergammaglobulinemia, reduced the development of a wide spectrum of autoantibodies, and restrained kidney inflammation, which coincided with the decreased transcription of IFN-stimulated genes (15). This finding is consistent with an earlier report that an anti-IFNα/β receptor-blocking antibody had a protective effect only in young BXSB mice (49). Similarly, a prophylactic IFN receptor blockade in young MRL-*Fas^lpr^* mice prevented the escalation of anti-RNP autoantibody titers and proteinuria transiently (49). Although the molecular entity that triggers IFN-I response in polygenic lupus-prone mice is unknown, the amyloid-induced autoimmunity explicitly illustrates that the activation of the pDC-IFN-I pathway can activate B cells, break immune tolerance, and induce antinuclear antibodies (41).

However, neither IFN-I nor pDCs are required for the regular antibody response to foreign antigens (13, 41, 50). Thus, the way in which the pDC-IFN-I axis promotes the positive selection, affinity maturation, and expansion of autoimmune B cells is enigmatic. The germinal center (GC) represents a unique lymphoid microenvironment where antigen-reactive B cells are expanded and diversified (51–53). Prevalent in the spleen of many lupus-prone mice, spontaneous GCs are crucial for the generation of autoantibodies (54–56). Interestingly, *Tcf4*-haplodeficient B6.*Sle1.Sle3* mice displayed reduced spontaneous GC reaction and a decreased GC-associated gene expression signature (14). Similarly, B6.Nba2 mice with depleted pDCs had significantly fewer GCs, as well as fewer follicular helper T cells and plasma cells, a phenomenon only observed in DT-treated young mice (16).

However, B cells other than GCs also constitute autoreactive B-cell compartments in a number of lupus models (57, 58). The overexpression of IFN-α *in vivo* sustained the proliferation of B cells and stimulated the expansion of short-lived plasmablasts, suggesting that the IFN-I-mediated autoimmune B-cell response had a non-GC origin (59). Age-associated B cells (ABCs), which lack CD21 and CD23 expression but express myeloid cell-specific markers, are stimulated by TLR7 activation and have an increased presence in lupus-prone mice (60–62). The findings from studies of BXSB, NZB, and B6-Fas*^lpr^* mice implied that ABCs diminish as a consequence of pDC ablation (13, 15). In contrast, the spleens of BXSB mice after DT injection contained increased numbers of marginal zone and transitional T1 B cells (15). Driven by *Tlr7* overexpression, T1 B cells undergo significant expansion and proliferation and are reportedly responsible for the production of autoantibodies in *Tlr7*-tg mice (57). Therefore, the way in which pDCs differentially regulate ABC and T1 B subsets and the relative contribution of specific B-cell subsets in the pathogenesis of lupus-prone mice remain to be elucidated. Furthermore, whether *Tcf4* haplodeficiency similarly impacts the T1 B-cell subset in the *Tlr7*-tg model is unclear.

In addition, pDCs may mediate emergency myelopoiesis, as indicated by the abated expansion of myeloid cells in *Tcf4*^+/−^*Tlr7*-tg mice (63). A previous study found that pDCs in the bone marrow (BM) of *Tlr7*-tg mice constitutively express IFN-I, which was hypothesized to drive the proliferation of Sca-1^+^ granulocyte/macrophage progenitors and subsequent expansion of peripheral myeloid cells (64). Whether the curbed myeloproliferation is mediated by the compromised IFN-I expression from BM pDCs remains to be determined. Of note, the normalization of the myeloid cell compartment was not reported in BXSB mice, which also harbor duplicated *Tlr7*, after continuous DT treatment (15). The reason for this discrepancy is unclear, but transgenic mouse lines knowingly expressing DTR in cDC lineage reportedly have depletion of additional cell types and elicit neutrophilia and monocytosis upon DT injection (65, 66). Nevertheless, IFN-I^+^ pDCs may functionally affect hematopoietic cells in the BM of mice with lupus. Indeed, pDCs from adult MRL-*Fas^lpr^* mouse BM transcribe IFNα and likely hinder the survival of B-cell progenitor cells (67).

Altogether, these recent studies are exceedingly significant and informative. The findings from diverse lupus models converge on the profound effect of pDCs toward humoral autoimmunity in general; while at the same time raise additional questions. Further detailed analysis is needed to determine whether pDCs differentially promote autoimmune B-cell responses in certain genetic and cellular contexts.

## Clinical Implications

Recent findings in lupus models about the pivotal function pDCs have in lupus pathogenesis support the tactic of therapeutically targeting pDCs in humans with SLE. TLR activation in pDCs under systemic autoimmune conditions hampers the effect of glucocorticoid treatment (68). In lupus-prone mice, pDCs seem to be more sensitive to Bcl2 antagonism, whose effectiveness could probably be improved with the addition of glucocorticoid therapy (69).

In their search for an effective treatment for SLE, researchers have developed numerous schemes to neutralize IFN-I or interfere with the function of IFNα/β receptor (8, 70, 71). Strategies to selectively deplete pDCs or suppress their IFN-I response should also help alleviate lupus (3). Given the predominant role of the pDC-IFN-I pathway in the early stages of lupus development, IFN-I and pDC blockade should be administrated to patients with relatively low disease scores. More detailed mechanistic studies will provide insight into disease-specific processes and enable innovation in SLE treatments.

## Conflict of Interest Statement

Yihong Yao is an employee of Cellular Biomedicine Group Inc. The other co-authors declare that the research was conducted in the absence of any commercial or financial relationships that could be construed as a potential conflict of interest.
